# Testing the Identical Effect on Predicted and Actual Memory Through Pictorial Stimuli

**DOI:** 10.1027/1618-3169/a000646

**Published:** 2025-06-11

**Authors:** Miri Besken, Gizem Filiz

**Affiliations:** ^1^ Department of Psychology, Bilkent University, Ankara, Turkey; ^2^ Department of Psychological & Brain Sciences Washington University in St. Louis, MO, USA

**Keywords:** identical effect, judgments of learning (JOLs), processing fluency, beliefs

## Abstract

**Abstract:** People tend to predict better memory for identical word pairs (e.g., DOG–DOG) than related ones (e.g., DOG–CAT), despite remembering related pairs more accurately—a phenomenon known as the *identical effect*. Across three experiments, we examined whether this illusion extends to pictorial materials and investigated the roles of processing fluency and a priori beliefs. Participants studied image pairs that were identical, exemplars, related, unrelated, or rotated (in Experiment 3). After each pair, they made judgments of learning (JOLs), and memory was later tested by a cued four-alternative forced-choice (4-AFC) recognition test. Consistently, identical image pairs received higher JOLs than related ones, despite equivalent or poorer recall. Identical pairs were also identified more quickly, reflecting greater processing fluency. However, identification speed did not consistently predict JOLs, suggesting that processing fluency alone cannot explain the illusion. These findings indicate that both processing fluency and beliefs influence JOLs, with beliefs about the pair types playing a central role.


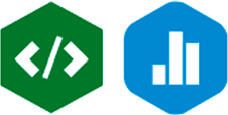


Metamemory refers to one's cognitive awareness and understanding of their own memory processes, encompassing a range of cognitive functions, including monitoring, control, and regulation (Dunlosky et al., 2016). Studies have shown that individuals can typically assess their own learning progress accurately (Hertzog et al., 2003; Rhodes, 2016). However, under certain circumstances, participants systematically fail to monitor their memory performance, leading to discrepancies between predicted and actual memory performance (Besken, 2018; Besken et al., 2019; Koriat & Bjork, 2005; Rhodes & Castel, 2008). These discrepancies, or metacognitive illusions, can result in suboptimal learning (Tullis & Benjamin, 2011). Therefore, accurate monitoring of one's memory is essential for efficient remembering. One widely used method for evaluating how individuals monitor their memory during the encoding process is through Judgments of Learning (JOLs). In this method, participants provide memory predictions for each trial during the learning phase for their performance in a subsequent memory test (see Rhodes, 2016, for a review).

One of the widely studied paradigms in the JOL literature is the semantic relatedness of word pairs (Mueller et al., 2013). In a seminal study, Arbuckle and Cuddy (1969) assessed participants' ability to correctly predict their memory for word pairs that have strong semantic relatedness (e.g., BREAD-LOAF) or weak semantic relatedness (e.g., UGLY-RUDE), showing that the semantic relatedness of word pairs is a strong predictor of both actual and predicted memory performance. Subsequent to this pioneering study, numerous studies have consistently shown that as semantic relatedness increases, both actual memory performance and metacognitive memory judgments during encoding increase (i.e., Hertzog et al., 2003; Koriat & Bjork, 2005; Maxwell & Huff, 2021; 2024; Mueller et al., 2013). Interestingly, a variation on the semantic relatedness phenomenon is the identical effect, in which participants assign higher memory predictions to identical (e.g., DOG–DOG) than related pairs (e.g., CAT–DOG), despite the lower memory performance for identical than related words (Castel et al., 2007) or equivalent memory performance for both (Mueller et al., 2016). The current study aims to test the underlying mechanisms for the emergence of the identical effect by using pictorial materials.

## Theory-Based and Experience-Based Processes in Identical Effect

The identical effect was initially put forward by Castel et al. (2007). In this study, participants were exposed to strongly related pairs (e.g., MILK–COW), weakly related pairs (e.g., MILK–JUICE), unrelated pairs (e.g., MILK–BOOK), and identical word pairs (e.g., MILK–MILK) in a within-subjects design, followed by JOLs after each trial. Subsequently, participants underwent a cued recall procedure to test their memory for the pairs. The results revealed that participants consistently assigned higher JOLs to identical than related word pairs during encoding, despite the lower recall for identical than related pairs at retrieval, producing a double dissociation between actual and predicted memory performance. This finding has been replicated in subsequent studies (Halamish & Undorf, 2023; Mueller et al., 2016). Moreover, Castel et al. (2007) found that participants’ predictions for identical words exceeded their actual memory performance, demonstrating an illusion of competence (Koriat & Bjork, 2005), with identical words producing higher JOLs and lower memory performance than their predictions.

The cognitive mechanisms underlying this effect are controversial and stem from a broader body of literature that explores the extent to which JOLs rely on *experience-based* versus *theory-based cues*. Koriat's multiple cue utilization framework (1997) posits that individuals' memory predictions are influenced by various factors such as the inherent characteristics of the stimuli (intrinsic cues), the presentation format, and the operations carried out by the participant on the stimuli (extrinsic cues), and the participant's interaction with the stimuli (mnemonic cues). Consequently, when making JOL ratings, participants may interact with the stimuli through two distinct processes*: Experience-based cues* involve participants' subjective feelings during the encoding task. The application of these subjective experiences to JOLs typically occurs automatically, often without the participant's conscious awareness. One cue that is frequently used is processing fluency. To explain, if participants experience more difficulty while processing certain items, they are likely to assign lower JOLs to these items as compared to those that are easier to process (Kornell et al., 2011). Typically, processing fluency is measured through various measures, such as subjective ratings of ease (Ardıç & Besken, 2023; Forster et al., 2013; Koriat et al., 2014), self-paced study time (Hertzog et al., 2003; Son & Metcalfe, 2000; Undorf & Erdfelder, 2011, 2015), and identification speed (Besken, 2016). On the other hand, *theory-based cues* refer to participants' pre-existing beliefs regarding the potential effects of experimental manipulations on memory. In these instances, participants consciously choose to apply an analytic theory based on specific characteristics of the study materials and how memorable they are expected to be due to those characteristics. For instance, if participants hold an a priori belief that perceptually similar materials are easier to remember, they will likely assign higher ratings to these items than perceptually dissimilar ones (Dunlosky et al., 2015; Mueller & Dunlosky, 2017; Mueller et al., 2013). Theory-based cues and their underlying processes are measured through scenarios of experimental manipulations (Ardıç & Besken; 2023; Besken, 2016; Besken et al., 2019; Mueller et al., 2014, 2016), pre-JOLs (Castel, 2008; Witherby & Tauber, 2017), or implantations of beliefs about the manipulation before the experiment starts (Mueller & Dunlosky, 2017). Typically, the findings show that participants use both experience-based and theory-based cues to produce JOLs. However, the extent to which participants use each of these cues tends to vary across different types of manipulations (Frank & Kuhlmann, 2017).

The identical effect has also been discussed within the framework of experience- and theory-based cues. Castel et al. (2007) contended that the identical effect relies on processing fluency, an experience-based cue because when participants were allowed to control the pace of their study time for each pair, they spent the least time on identical pairs. Accordingly, Castel et al. (2007) argued that the increased processing fluency associated with identical pairs may serve as the underlying mechanism driving this identical effect illusion. They claimed that semantic relatedness is essential for the effect to emerge, but identical pairs also benefit from the increased perceptual similarity (or sameness) for identical than related word pairs.

In contrast, a subsequent study by Mueller et al. (2016) provided evidence that the identical effect may not be mediated by processing fluency. In this study, participants were exposed to three types of pairs: identical matched pairs (e.g., CaT–CaT) with both perceptual and conceptual similarity (or sameness), identical mismatched pairs (e.g., CaT–cAt) with lower perceptual similarity but equivalent conceptual similarity, and related pairs lower in conceptual similarity and no perceptual similarity (e.g., CaT–MeOw). The results revealed that participants assigned higher JOLs to identical matched pairs than to identical mismatched pairs, but the self-paced study times did not significantly differ across these two conditions. This finding suggests that the effect is not contingent on processing fluency, as evidenced by the similar study times allocated to both identical matched and mismatched pairs. Further experiments through the presentation of scenarios about the experiment also showed that participants also carry a belief that identical pairs will produce higher memory performance than related pairs (Exp 3). Moreover, when participants made their JOLs before exposure to the word pair, they produced higher JOLs for identical than related pairs (Exp 4). Thus, Mueller et al. (2016) concluded that the identical effect is mediated by theory-based, a priori beliefs rather than processing fluency because participants hold these beliefs before or even without exposure to the word pairs themselves.

## Current Study: Investigating the Identical Effect Through Pictorial Materials

The primary objective of the current study is to gain a deeper understanding of the identical effect through using pictorial materials. While existing literature predominantly explores the identical effect with written and verbal materials (Castel et al., 2007; Halamish & Undorf, 2023; Mueller et al., 2016), employing pictorial materials offers a more nuanced exploration of the contributions of perceptual and conceptual similarities to this phenomenon. Building on Mueller et al.'s (2016) work, which utilized matched (e.g., CaT–CaT) and mismatched (e.g., CaT–cAt) verbal stimuli to examine the impact of perceptual similarity on the identical effect, our study leverages the richer representational depth of pictorial materials. For example, in Mueller et al. (2016), participants may have had difficulty processing the nuance between the perceptually mismatched version of “CaT” and “cAt,” which was reflected in statistically similar study times for identical matched and mismatched items. In this study, participants viewed image pairs that were identical (e.g., two identical cats), similar (e.g., two different cats), related (e.g., a cat and a dog), or unrelated (e.g., a cat and a table). Similar pairs allowed us to isolate perceptual similarity: They are more alike than related pairs but less so than identical ones. Because similar pairs feature two distinct cat images, differences between them become more noticeable, despite shared features such as four legs, whiskers, and tails. In contrast, perceptual overlap is more immediately evident in identical pairs. Compared to related pairs, similar pairs show subtler differences, highlighting the stronger perceptual gap in related pairs.

Distinguishing our approach from verbal stimuli, which predominantly engage linguistic and auditory brain regions, pictorial material involves processing in the visual areas (Schlochtermeier et al., 2013). Dewhurst and Conway (1994) contended that pictures are encoded with richer sensory-perceptual details and access this shared semantic code more readily than words. In contrast, words tend to activate phonemic and orthographic processes before accessing semantic codes (Dewhurst & Conway, 1994; Nelson & Borden, 1977). Consequently, processing verbal materials may rely on language-specific processes, resulting in representations with limited sensory-perceptual details. Moreover, Amit et al. (2009) argued that pictures closely resemble their physical referents and undergo similar perceptual processes as their referent objects, whereas words are more detached from their referent objects. Thus, the perceptual analysis of pictures may evoke more specific and concrete representations compared to words. This analysis increases the likelihood that participants process the perceptual information more effectively and enables a more detailed examination of perceptual nuances between image pairs, as in the current study.

The general procedure for all three studies involved presenting participants with identical, similar, related, and unrelated images in an experimenter-paced encoding phase, followed by JOLs after each stimuli pair. During testing, participants were provided with the first image of the pair and asked to remember the second image from one of four options: identical, similar, related, or unrelated. Experiment 1 used this basic design. Experiment 2 additionally required participants to indicate whether the presented pairs were identical, similar, related, or unrelated during encoding to measure processing fluency. Experiment 3 replaced unrelated pairs with rotated pairs to better isolate the effects of perceptual similarity. Unlike unrelated pairs, rotated pairs maintain both conceptual and object identity while introducing a perceptual transformation. This allows for a more precise examination of whether perceptual similarity alone contributes to the identical effect. We hypothesized that if the perceptual similarity is the driving force behind inflated JOLs for identical pairs, then similar pairs should receive higher JOLs than related pairs because the perceptual similarity is higher across similar than related pairs. Moreover, the processing fluency, operationalized as the speed to process the type of relationship between the pairs (i.e., similar or related), should predict JOLs if the effect is contingent on experience-based cues.

## Experiment 1

It was shown that identical word pairs receive inflated JOLs while leading to poorer memory performance for those items (Castel et al., 2007; Mueller et al., 2016). However, the effect of the identical items on JOLs has not been studied in different modalities. Thus, the current study aimed to investigate the relationship between JOLs and actual memory for four different types of image pairs to ensure that the identical effect could be replicated through pictorial materials. Similar to the study of Castel et al. (2007), identical, related, and unrelated pairs were used. However, related pairs were classified into two distinct categories. One of the categories was exemplars of the same object (e.g., two different photos of cupcakes). These objects are conceptually the same and perceptually similar, but they are not identical. The other category was the related pairs, which are conceptually related, but perceptually dissimilar, so if the identical effect is caused by the mere perceptual similarities, participants should produce higher JOLs for similar than related pairs. Thus, we hypothesized that identical pairs should lead to the highest JOLs with a gradual decrease in JOLs for similar, related, and unrelated items. In contrast, actual memory performance should be higher for similar and related pairs, followed by identical and unrelated ones, in line with previous research.

### Method

#### Participants

Twenty-four native Turkish speakers aged between 18 and 30 years were recruited to experiment from Bilkent University either for course credit or voluntarily. Even though their demographic data was not collected for this or subsequent experiments, participants mostly consisted of university students who are attending experiments to collect credit for a general education course. The results of an a priori power analysis (G*Power version 3.1.9.7; Faul et al., 2007) showed that a sample size of *N* = 24 is sufficient to achieve 80% power for detecting a medium effect (*f* = .25) at a significance level of α = .05 in a single-factor repeated measures ANOVA. Prior studies have also used similar sample sizes (Castel et al., 2007; Experiment 1; Mueller et al., 2016).

#### Materials and Design

Images were sourced from the visual database named Pool of Pairs of Related Objects (POPORO), tested, and validated for semantic (un)relatedness through both behavioral ratings and ERP findings (see Kovalenko et al., 2012 for further details). This database includes photos of daily objects such as utensils, vehicles, animals, and plants, all presented on a white background in 400 × 400 pixels. Each prompt item (e.g., a carrot image) in the database is paired with one related (e.g., a rabbit image) and one unrelated target item (e.g., a pump image).

The experiment was designed as a within-subject study with four levels of pair types: identical, similar, related, and unrelated. Forty images from POPORO were selected as prompt images and were randomly divided into four groups of 10 items each. These groups were paired with target images that were either identical, related, unrelated, or similar to the prompt. Identical item pairs were created by repeating the prompt image as the target image (e.g., carrot photo 1–carrot photo 1). Related pairs were created using the related target items in the POPORO database. These items were conceptually related to the prompt image but perceptually dissimilar (e.g., carrot photo 1–rabbit photo). In other words, the related pairs exhibited a conceptual relationship between the prompt and target images, similar to the related word pairs described by Castel et al. (2007), such as an image of a computer paired with a keyboard. Unrelated target items were again taken from the POPORO database, consisting of images having no semantic or perceptual relationship with the prompt image (e.g., carrot photo 1–pump photo). Similar pairs consisted of images that are exemplars of the same item category, such as two different carrots (e.g., carrot photo 1–carrot photo 2). As no exemplars of the same object were available in the database, additional images were chosen from a free image search on Google. They were chosen based on their distinct variations within the same category, such as different types of candles or various styles of hats. While these items may differ in color, shape, or design, they still fundamentally represent the same concept—a candle or hat. For instance, one candle may be tall and slender while another is short and wide, yet both serve the same purpose and belong to the same class of objects. The variations highlight the exemplars within a single category, even though their core identity remains unchanged. The image pairs in similar conditions were equated on various image characteristics, such as size and quality. Moreover, we pilot tested the similarity of objects with a group of participants to ensure that they named similar objects as referring to the same concept.

The pair type was counterbalanced across participants so that the prompt item was equally matched with target items in different pair types. For instance, if one participant saw identical photos of the carrot as both the prompt and the target (identical condition), another participant saw the carrot as the prompt and the rabbit as the target item (related condition). An equal number of participants were recruited for each counterbalance condition (six per condition). As the database only contains standardized images for the first item of the pair, the prompt item remained the same for all participants, but the target photo varied. Additionally, two image pairs were included for practice, and four pairs each for primacy and recency effects.

#### Procedure

All participants were tested individually in a lab room with a computerized setting through E-Prime 2.0 software (Schneider et al., 2002). The experiment consisted of three phases: encoding, distractor, and testing phases. Before the study, participants were told they would be exposed to different image pairs. They were specifically explained that in a subsequent memory test, they would be prompted with the image on the left of the screen and asked to remember the one on the right in a recognition test. They were instructed to provide JOLs after each trial by indicating how likely they were to remember the second item (on the right) of the pair if they were given the first item (on the left) on a subsequent memory test. JOLs ranged from 0 (I will definitely not remember these items) to 100 (I will definitely remember these items) and were self-paced.

**Figure 1:**
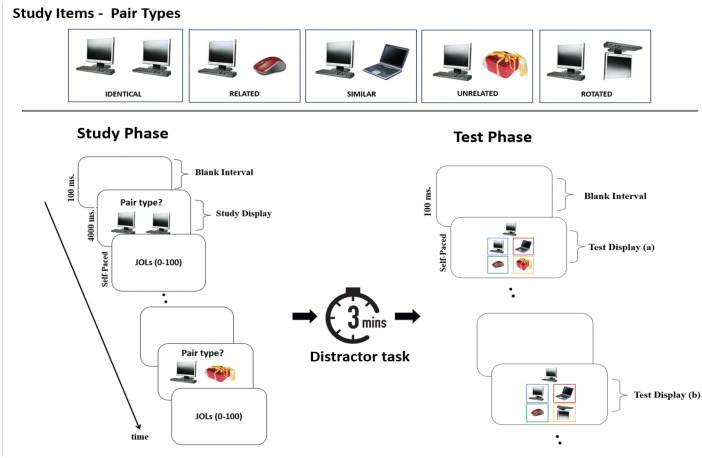
General experimental display across three experiments. Unrelated pairs used in the testing phase of Experiments 1 and 2 (Test Display a) were switched to the rotated pairs in Experiment 3 (Test Display b). The images used in this illustration are not the real images used in the experiments in order to avoid copyright infringement issues.

As shown in Figure 1, during encoding, each trial started with a blank screen for 100 ms, followed by the presentation of an image pair, which participants viewed passively for 4,000 ms. JOL screen followed the encoding screen, during which participants typed a number between 0 and 100 and pressed the ENTER key to proceed to the next trial. Participants completed two practice trials, followed by four trials for primacy, 40 critical trials, and four recency trials. Practice, primacy, and recency trials were not included in the testing phase.

In the distractor phase, participants solved basic math problems (e.g., 50 − 28 = ?) for 3 minutes. In the testing phase, participants were presented with the prompt picture at the top center of the screen with four options as identical, similar, related, or unrelated to select as in a cued four-alternative forced-choice (4-AFC) recognition test (Figure 1 Test Display a) and were asked to select the target picture presented at encoding. For each option, images were framed with certain colors (i.e., green, red, blue, and yellow) matching with the key color on the keyboard marked with stickers of those colors. There was no time restriction for the testing phase.

### Results

**Figure 2:**
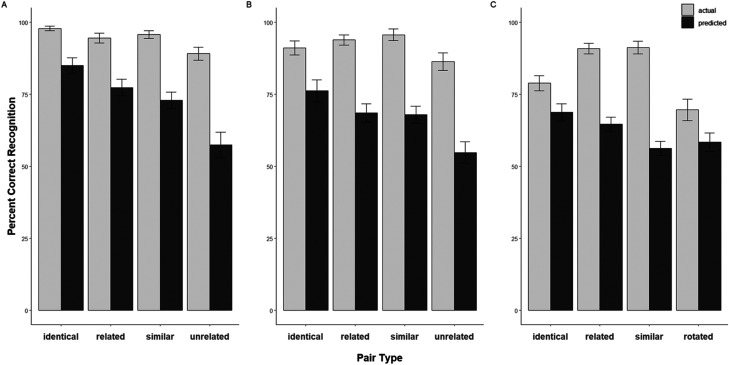
Mean predicted (JOL) and actual percent correct recognition as a function of the four types of image pairs across three experiments. Error bars represent standard errors of the mean. Panels A, B, and C represent Experiments 1–3, respectively. The actual recall was calculated by multiplying the percent correct recognition by 100 to provide a comparison between JOLs (predicted) and percent correct (actual) recognition.

The data were analyzed in R using the Tidyverse (Wickham et al., 2019), lme4 (Bates et al., 2015), and lmerTest (Kuznetsova et al., 2017) packages. The data and R-scripts for all analyses are publicly available online at the Open Science Framework (OSF; https://osf.io/mxgsd/). Table 1 presents the descriptive statistics for Experiments 1–3. Figure 2A shows the mean performance rate for predicted memory responses (JOLs) and actual mean recognition percentages across the four pair types (i.e., identical, similar, related, and unrelated). For all analyses, the alpha level was set at .05. For all pairwise comparisons, Bonferroni correction was used.

**Table 1:** *M* and *SD* of the judgments of learning and cued 4-AFC recognition performance across types of image pairs for Experiments 1–3

Pair types	Experiment 1 (*N* = 24)				Experiment 2 (*N* = 24)				Experiment 3 (*N* = 26)			
	Judgments of learning (predicted)		Percent correct recognition (actual)		Judgments of learning (predicted)		Percent correct recognition (actual)		Judgments of learning (predicted)		Percent correct recognition (actual)	
	*M*	*SD*	*M*	*SD*	*M*	*SD*	*M*	*SD*	*M*	*SD*	*M*	*SD*
Identical	85.06	13.37	97.92	4.15	76.25	18.85	91.18	11.86	68.78	15.01	78.91	13.14
Related	77.39	13.95	94.58	8.33	68.55	15.94	93.90	8.79	64.66	12.53	90.90	9.20
Similar	72.92	14.03	95.83	6.54	67.94	14.61	95.70	9.92	56.30	12.51	91.28	11.05
Unrelated/rotated^a^	57.47	21.71	89.17	11.00	54.85	18.42	86.39	14.71	58.43	16.37	69.63	19.14
*Note*. ^a^Unrelated pairs are switched to rotated pairs in Experiment 3.												

#### JOL Ratings

Table 1 shows the mean JOL ratings across the four pair types (identical, similar, related, and unrelated). These means were submitted to a single-factor repeated measures ANOVA and revealed significantly different JOLs for each pair type, *F*(3, 69) = 24.38, *MSE* = 133.20, *p* < .001, η_*p*_^*2*^ = .51. Pairwise comparisons for each pair type demonstrated that identical pairs received significantly higher JOL ratings than similar and unrelated pairs [*identical* versus *similar: t*(23) = 5.18, *d* = 1.06, *p* < .001; *identical* versus *unrelated*: *t*(23) = 5.74*, d =* 1.17*, p* < .001], but did not significantly differ from related pairs, *t*(23) = 2.51, *d* = 0.51, *p* = .116. JOLs for similar and related pairs did not significantly differ from each other, *t*(23) = 2.67, *d* = 0.55, *p* = .082. Unrelated pairs produced significantly the lowest JOL rating compared to all other pair types [*related* versus *unrelated*: *t*(23) = 6.10, *d* = 1.25, *p* < .001; *similar* versus *unrelated*, *t*(23) = 3.96, *d* = 0.81, *p* = .004].

#### JOL RT

For encoding, the median response latency for completing JOLs was calculated separately for all pair types for each participant. These means were submitted to a single-factor repeated measures ANOVA and revealed no significant differences across conditions, *F*(3, 69) = 1.12, *MSE* = 160,816.17, *p* = .347, η_*p*_^*2*^ = .046.

#### Cued 4-AFC Recognition Accuracy

Table 1 shows the accuracy rate for the cued 4-AFC recognition performance of participants across the four pair types (identical, similar, related, and unrelated). The single-factor repeated measures ANOVA showed that the accuracy rate significantly differed among pair types, *F*(3, 69) = 7.46, *MSE* = .004, *p* < .001, η_*p*_^2^ = .24. Pairwise comparisons indicated that the accuracy rate of identical, similar, and related pairs did not significantly differ from each other, [*similar* versus *identical*: *t*(23) = 1.42, *d* = 0.29, *p* = 1.00; *related* versus *identical*: *t*(23) = 2.00, *d* = 0.41, *p* = .344; *similar* versus *related* pairs: *t*(23) = 0.72, *d* = 0.15, *p* = 1.00]. However, identical pairs' accuracy rate was higher than unrelated ones, *t*(23) = 3.98*, d* = 0.81, *p* = .004. Unrelated images’ accuracy rate did not significantly differ from related ones, *t*(23) = 2.72, *d* = .55*, p* = .074, and similar ones, *t*(23) = 2.80*, d* = 0.57, *p* = .061.

### Discussion

Experiment 1 showed that the pair type had a significant impact on JOLs even though the actual recognition was not influenced to the same degree. That is, identical, similar, and related pairs were recognized at similar rates, while unrelated pairs were recognized at a lower rate. Although participants provided the highest JOLs for identical pairs, their actual recognition was not different across identical, similar, and related pairs. These findings are partly in line with the original study of Castel et al. (2007) and Mueller et al. (2016). There was a difference between predicted and actual memory performance for identical pairs. However, the recognition rates for related items were not higher than identical ones despite the higher JOLs given to identical items. A pattern similar to the current study was also found in Halamish and Undorf (2023). Moreover, the critical comparison for JOLs between similar and related pairs shows a significant advantage for related over similar pairs. Thus, this rules out the possibility that the inflated JOLs for perceptually more similar pairs originate from perceptual similarity. If that were the case, similar pairs should have received higher JOLs than related pairs. Moreover, as an indirect measure of how quickly participants perceived the items, JOL response latencies revealed no differences across conditions. This argues against an account of perceptual fluency because participants should provide JOLs slower if this were the case. More direct measures are employed in Experiments 2 and 3 to assess this further.

For this study, 40 image pairs were used. This quantity is similar to those used in previous studies (e.g., Mueller et al. (2016) used 36-word pairs, and Castel et al. (2007) used 48-word pairs). However, the memory boosting effect of visual materials on a cued 4-AFC recognition test may have caused a ceiling effect in the current design, producing 90% accuracy in recognition performance even for unrelated pairs. This is predictable since items studied as images generally lead to higher recognition performance than words (Mintzer & Snodgrass, 1999). The subsequent two experiments increase the number of image pairs in each condition used during the encoding phase to handle this issue.

## Experiment 2

Experiment 2 is a modified version of Experiment 1 with two changes in the procedure: First, the number of trials increased from 40 to 80 at encoding to eliminate the ceiling effect obtained for actual memory performance in Experiment 1. The second modification involved a more conceptual question. It has been shown that items that are processed faster also elicit inflated JOLs (Koriat & Ma’ayan, 2005). Accordingly, Castel et al. (2007) employed a self-paced study method to investigate the identical effect and processing fluency relationship and found that participants allocated less time and assigned higher JOLs to identical than related item pairs. They inferred that the inflated JOLs for the identical effect could be explained by processing fluency or ease of processing, which is reflected in the study time. Mueller et al. (2016) found similar results, but further analyses revealed that the relationship is not mediated by the study time. The identical effect may depend both on conceptual and perceptual similarities of the item pairs; however, as Mueller et al. (2016) also contend, self-paced study times may not successfully distinguish conceptual and perceptual aspects, and they advised utilization of different fluency measures to examine this relationship. For example, Mueller et al. (2013) used a lexical decision task to measure processing fluency in a relatedness study.

In Experiment 2, both the accuracy and speed of participants in identifying pair types were examined during the encoding phase of the experiment. The goal was to further disentangle the perceptual and conceptual components of this task. Participants were instructed to quickly and accurately categorize the pairs they encountered as identical, similar, related, or unrelated. By measuring how quickly participants identified the different types of pairs, processing fluency for the type of relationship between cue and target may be assessed more directly. We hypothesized that the identical pairs should receive the highest JOLs and be identified in the shortest amount of time during encoding than other types of pairs. However, this trend should not be reflected in the actual memory performance.

Multilevel mediational analyses were also performed to see whether identification RT mediates the effect of pair type on JOLs. The direct effects in these mediation analyses indicate whether pair type has a direct impact on JOLs, while the indirect effects reveal whether this influence operates through reaction time. If perceptual fluency is the primary factor influencing the effect, JOLs should be highest for identical pairs, followed by similar, and related pairs, reflecting a gradual decline in perceptual similarity. Additionally, identification time should serve as a mediator in the relationship between the experimental manipulation and JOLs, with faster identification of identical pairs leading to higher JOLs through reduced processing time, leading to significant indirect effects. Conversely, if the identical effect is driven solely by metacognitive beliefs, identification time should not mediate the relationship between pair type and JOLs. In this case, the mediation analysis should reveal significant direct effects but no significant indirect effects (see Undorf et al., 2017, Yang et al., 2019 for similar logic and analyses).

### Method

#### Participants

As in Experiment 1, 24 native Turkish speakers between the ages of 18 and 30 from Bilkent University participated in the study. They received course credits in exchange for their participation, or they participated voluntarily.

#### Materials, Design, and Procedure

Experiment 2 is similar to Experiment 1, with the exception of the following differences: First, the practice items were increased from two to eight (two trials for each pair type), and the number of primacy and recency items were held constant. The number of critical trials was increased from 40 to 80. The pair types were counterbalanced across participants, as in Experiment 1.

In Experiment 2, participants were asked to identify the type of object relationship among pairs (i.e., identical, similar, related, or unrelated) by pressing the designated keys (A, S, D, or F) on the keyboard during encoding. To ensure a clear understanding, the experimenter provided examples of each pair type, illustrating what similar and related pairs look like and their conceptual implications. Following this, participants were shown example trials where they needed to identify the pair type and provide their JOLs on the computer. The experimenter encouraged questions and provided any needed clarifications. Once ready, participants began the task by pressing the SPACE key on the keyboard. During each encoding trial, regardless of whether they responded or not, they continued to see the image pairs; they were not allowed to proceed to the JOL screen before 4,000 ms. Once participants identified the pair type by pressing the allocated button on the keyboard, a gray frame appeared around the image pair to inform them that their response was recorded. After the encoding phase, they proceeded to the distractor task and completed the recognition test as the last phase of the experiment. All the distractor and test procedures were identical to Experiment 1.

### Results

#### Identification Accuracy

During encoding, the mean identification rates for pair types were computed (see Table 2) and submitted to a single-factor repeated measures ANOVA. This analysis revealed that there are significant differences in identification accuracy between pair types, *F*(3,69) = 28.17, *MSE* = .005, *p* < .001, η_*p*_^*2*^ = .55. Pairwise comparisons showed that identical pairs were identified more than related and similar pairs [*identical* versus *related*: *t*(23) = 10.20, *d* = 2.07, *p* < .001, *identical* versus *similar*: *t*(23) = 4.60, *d* = 0.94, *p* < .001]. For unrelated pairs, there was a numerical trend, but no significant differences, *t*(23) = 2.81, *d* = 0.57, *p* = .06. The identification rate of similar and related pairs did not differ, *t*(23) = 2.26, *d* = 0.46, *p* = .202. The identification rate was higher for unrelated pairs compared to related and similar pairs [*related* versus *unrelated*, *t*(23) = 7.72, *d* = 1.58, *p* < .001; *similar* versus *unrelated*, *t*(23) = 3.38, *d* = 0.69, *p* = .015].

**Table 2:** *M* and *SE* of the estimate for identification reaction time and accuracy across types of image pairs in Experiments 2 and 3

Pair types	Experiment 2 (*N* = 24)				Experiment 3 (*N* = 26)			
	Identification RT		Identification accuracy		Identification RT		Identification accuracy	
	*M*	*SE*	*M*	*SE*	*M*	*SE*	*M*	*SE*
Identical	1,131	56	.99	.004	1,291	63	.99	.004
Related	2,035	64	.82	.017	1,840	111	.80	.017
Similar	1,901	74	.88	.015	2,065	88	.77	.018
Unrelated/rotated^a^	1,776	70	.96	.008	1,341	59	.96	.008
*Note*. ^a^Unrelated pairs are switched to rotated pairs in Experiment 3.								

#### Identification RTs

For encoding, the median first keypress response latency (identification response times [RTs]) was calculated separately for all pair types for each participant (see Table 2), excluding trials in which participants misidentified the pair types or did not respond at all (exclusion rate = 8.75%), and the means of the medians were submitted to a repeated measures ANOVA. The analysis shows that the pair type had a significant effect on first keypress latency, *F*(3,69) *=* 80.13, *MSE* = 48,098.85, *p* < .001, η_*p*_^*2*^ = .78. Pairwise comparisons showed that identical pairs were identified faster than all other pairs [*identical* versus *related: t*(23) = 15.2, *d* = 3.09, *p* < .001*; identical* versus *similar: t*(23) = 11.1, *d* = 2.26, *p* < .001; *identical* versus *unrelated: t*(23) = 12.4, *d* = 2.53, *p* < .001]. RT for similar and related pairs did not differ, *t*(23) = 2.08, *d* = 0.42, *p* = .293, while unrelated pairs were identified faster than related pairs, but not than similar pairs [*related* versus *unrelated: t*(23) = 4.10, *d* = 0.84, *p* = .003; *similar* versus *unrelated: t*(23) = 1.81, *d* = 0.37, *p* = .497].

#### JOL Ratings

For JOLs, all trials in which the participants misidentified the pair types during encoding or failed to enter a value between 0 and 100 for JOLs were excluded from the analyses (exclusion rate = 9.06%). Figure 2B shows the mean performance rate for JOLs and correct percentage recognition across the four encoding pair categories. The means were submitted to a single-factor repeated measures ANOVA, revealing significant JOL differences across pair types, *F*(3, 69) = 32.94, *MSE* = 57.44, *p* < .001, η_*p*_^*2*^ = .59. Pairwise comparisons for each pair type demonstrated that the identical pairs produced significantly higher JOLs than all the other pair types [*identical* versus *related*: *t*(23) = 3.24, *d* = 0.66, *p* = .022; *identical* versus *similar*: *t*(23) = 3.58*, d* = 0.73, *p* = .009; *identical* versus *unrelated*: *t*(23) = 7.79, *d* = 1.59, *p* < .001]. However, similar and related pairs did not significantly differ from each other, *t*(23) = .70, *d* = 0.14, *p* = 1.00. Unrelated pairs produced significantly lower JOLs than related and similar pairs [*related* versus *unrelated: t*(23) = 6.13, *d* = 1.25, *p* < .001; *similar* versus *unrelated*: *t*(23) = 6.27, *d* = 1.28, *p* < .001].

#### Conditional Cued 4-AFC Recognition^1^

Figure 2B shows the mean accuracy rate for the cued 4-AFC recognition performance of participants across the four pair types (identical, similar, related, and unrelated). The single-factor repeated measures ANOVA showed that the accuracy rate significantly differed among pair types, *F*(3, 69) = 8.26, *MSE* = .005, *p* < .001, η_*p*_^2^ = .26. Pairwise comparisons indicated that the accuracy rate of the identical pairs did not differ from any other pairs [*identical* versus *similar*: *t(*23) = 2.55, *d* = 0.52, *p* = .107; *identical* versus *related*: *t*(23) = 1.31, *d* = 0.27, *p* = 1.00; *identical* versus *unrelated*: t(23) = 2.28, *d* = 0.47, *p* = .193]. Similarly, related and similar pairs did not differ in accuracy either, *t(*23) = 1.02, *d* = 0.21, *p* = 1.00. The accuracy for unrelated pairs was significantly the lowest among all other pairs [*related* versus *unrelated: t*(23) = 3.10, *d* = 0.63, *p* = .03; *similar* versus *unrelated: t*(23) = 5.37, *d* = 1.10, *p* < .001].

#### Mediation Analysis

Mediation analyses were carried out by using the ‘boot’ package in R (Canty & Ripley, 2024) to perform bootstrapping and calculate bias-corrected and accelerated (BCa) confidence intervals (Efron, 1987; for the implementation of this method, see Davison & Hinkley, 1997). All models were specified within a multilevel mediation structure, with item type (predictor), encoding RT (mediator), and JOLs (outcome) measured at the trial level (Level 1), and random intercepts for participants modeled at Level 2. Indirect, direct, and total effects were estimated for each contrast using bootstrapped 95% bias-corrected and accelerated (BCa) confidence intervals based on 5,000 resamples. Using bootstrapping allows the calculation of indirect effects, even with small samples, by generating bias-corrected and accelerated confidence intervals (Shrout & Bolger, 2002).

The multilevel mediational analysis aimed to investigate how perceptual and conceptual similarities contribute to metacognitive judgments, in which encoding identification time (RT) served as a proxy for processing fluency. Crucially, item type was dummy coded using the “Similar” condition as the reference category, which represents a theoretically balanced midpoint. These items involve the same concept but different perceptual instances (e.g., two different chairs). This setup enabled more precise contrasts, identical versus similar pairs isolate the effects of perceptual similarity, while related versus similar pairs isolate conceptual similarity. Unrelated items were also included for exploratory completeness, although they do not directly inform the core perceptual-conceptual contrast and were not central to the primary hypothesis.

##### Identical Versus Similar Pairs

The indirect effect of the identical pairs (compared to similar), capturing the role of processing fluency, was statistically significant (β = 2.59, 95% CI [0.90, 4.41]), indicating that these items were encoded more fluently than similar items, and this fluency led to higher JOLs. The direct effect (β = 5.73, 95% CI [2.95, 8.71]) was also significant, reflecting belief-based expectations about memorability. The total effect was β = 8.32, 95% CI [5.83, 10.83]), and the proportion mediated was approximately 31% (95% CI [0.12, 0.56]). This pattern shows that both mechanisms jointly influence JOLs for identical items, with theory-based reasoning contributing more heavily.

##### Related Versus Similar Pairs

Related items compared to similar items, showed a negative and significant indirect effect (β = −0.50, 95% CI [−0.97, −0.18]), indicating they were processed less fluently than similar items, which in turn led to lower JOLs. At the same time, the direct effect was positive but nonsignificant (β = 1.26, 95% CI [−1.48, 3.88]). The opposite directions of the direct and indirect effects suggest a suppression effect (MacKinnon et al., 2000), where participants' theory-based processes may have boosted JOLs, but this was counteracted by the low processing fluency. As a result, the total proportion and proportion mediated are not meaningful in this condition and should not be reported (MacKinnon et al., 2000). This pattern suggests a conflict between belief-based and fluency-based signals, resulting in the cancellation of their effects.

##### Unrelated Versus Similar Pairs

Unrelated items showed a distinct pattern. The indirect effect was statistically significant but very small (β = 0.17, 95% CI [0.003, 0.48]), suggesting a minimal role for processing fluency. In contrast, the direct effect was large and negative (β = −13.35, 95% CI [−16.11, −10.69]), indicating that participants held strong beliefs about the difficulty or memorability of unrelated items. The total effect and the proportion mediated are not reported, as they are not interpretable in this analysis.

### Discussion

Experiment 2 provided evidence for two main points: First, identical pairs were identified fastest and given the highest JOLs among all pair types. Even though participants produced the highest JOLs for identical items, all pair types (except for unrelated pairs) produced null results in terms of memory accuracy, showing that the identical effect remains as a metacognitive illusion, both consistent with Experiment 1 and previous studies (Castel et al., 2007; Halamish & Undorf, 2023; Mueller et al., 2016).

Second, the study supports dual-process accounts of JOLs, highlighting contributions from both fluency-based and theory-based processes, with belief-based processes playing a dominant role. Evidence for this includes the finding that related and similar pairs took the longest to identify and did not differ in either identification RT or JOLs—possibly reflecting difficulty in distinguishing the type of relationship. Mediation analyses revealed that for identical items, both fluency and beliefs influenced judgments, although the belief-based reasoning explained about two-thirds of the effect. Identical pairs boosted confidence even when RT was controlled, suggesting a direct impact of beliefs about perceptual similarity on JOLs. For related items, a suppression effect emerged, indicating competing influences of fluency and beliefs, resulting in no net effect. For unrelated items, judgments were largely belief-driven, with participants relying on perceived item difficulty over subjective fluency. Overall, Experiment 2 suggests that perceptual similarity—greater in similar than related pairs—did not always meaningfully predict JOLs, and that fluency, as measured by identification RTs, played a relatively minor role, consistent with Mueller et al. (2016).

## Experiment 3

Even though using similar images is a useful methodology in disentangling the contributions of perceptual similarities to the identical effect, one can take perceptual similarity operationalization even further by using rotated objects. Rotated objects are both conceptually and perceptually the same as the target image; however, the rotation of the same object still produces a perceptual mismatch between the target picture and its rotated version. Identifying a rotated picture takes longer than an upright picture (Besken et al., 2019), so if the identical effect is a consequence of processing fluency, rotated pairs should take longer to identify than identical pairs and accordingly produce lower JOLs. At the same time, the rotation manipulation produces even a finer graded distinction between identical and similar pairs. Identification RT should be faster for rotated than similar pairs since rotated pairs have the exact same perceptual appearance and an orientational transformation, whereas similar pairs are perceptually less comparable. This should also be reflected in JOLs linearly if processing fluency of the perceptual similarities is indeed the source of the identical effect. Moreover, the mediational analyses should also reflect this linear relationship between processing time and JOLs.

Previous studies reveal that when presented alone, rotated objects do not typically produce higher memory performance than upright objects (Besken et al., 2019). Accordingly, we predicted that rotated pairs should not produce results different from those of identical pairs. Besides, similar and related pairs typically produce higher memory accuracy, in line with the findings of the classical identical effect (Castel et al., 2007).

### Method

#### Participants

Twenty-six students from Bilkent University, aged 18–30, participated in the experiment in exchange for course credits or voluntarily.

#### Materials, Design, and Procedure

The method and procedure used in Experiment 3 were the same as in Experiment 2. However, the following changes to the procedure were made: Unrelated pairs were replaced with rotated pairs, and participants were again shown four picture types: identical, rotated, similar, and related. Rotated images were created by rotating the images 180°. All items that were used in the experiment had one upright direction and were always presented on the right of the screen. All pair types were counterbalanced across participants. Unrelated pairs were completely excluded to lower the number of conditions for pairwise comparisons and also because Experiments 1 and 2 replicated the same finding: Unrelated pairs always resulted in the lowest JOL and poorest memory performance. The rest of the materials and the procedure were the same as in Experiments 1 and 2.

### Results

#### Identification Accuracy

During encoding, the identification rates for pair types were computed (see Table 2) and submitted to a single-factor repeated measures ANOVA. This analysis revealed significant differences in identification accuracy between pair types, *F*(3,75) = 22.58, *MSE* = .014, *p* < .001, η_*p*_^*2*^ = .47. Pairwise comparisons showed that identical pairs were identified more often than all other pairs [*identical* versus *related*: *t*(25) = 5.76, *d* = 1.13, *p* < .001; *identical* versus *similar*: *t*(25) = 7.13, *d* = 1.40, *p* < .001; *identical* versus *rotated*: *t*(25) = 2.98, *d* = 0.58, *p* = .038]. Rotated pairs were identified more often than similar and related pairs [*rotated* versus *related: t*(25) = 4.99, *d* = 0.98, *p* < .001; *rotated* versus *similar: t*(25) = 5.95, *d* = 1.17, *p* < .001]. However, the identification rate of similar and related pairs did not differ, *t*(25) = 0.74, *d* = 0.14, *p* = 1.00.

#### Identification RTs

The means of the median RTs (see Table 2) were submitted to a repeated-measures ANOVA, excluding invalid trials (11.92%), using the criteria in Experiment 2. The analysis yielded a significant effect on response latency, *F*(3,75) = 64.18, *MSE* = 58,871.78, *p* < .001, η_*p*_^*2*^ = .72. Pairwise comparisons showed that identical pairs were identified faster than other pairs [*identical* versus *related: t*(25) = 6.96, *d* = 1.36, *p* < .001, *identical* versus *similar: t*(25) = 11.8, *d* = 2.32, *p* < .001], except for the rotated pairs, *t*(25) = 2.29, *d* = 0.45, *p* = .185. Rotated pairs were identified faster than similar and related pairs [*rotated* versus *similar: t*(25) = 12.00, *d* = 2.36, *p* < .001; *rotated* versus *related: t*(25) = 6.59, *d* = 1.29, *p* < .001]. Similar and related pairs’ identification time did not differ *t*(25) = 2.70, *d* = 0.53, *p* = .074.

#### JOL Ratings

For JOLs, the same exclusion criteria were used as in Experiment 2 (exclusion rate = 12.35%). Figure 2C shows the JOL ratings across the four pair types. A single-factor repeated measures ANOVA revealed significantly different JOLs for each pair type, *F*(3, 75) = 8.00, *MSE* = 106.28, *p* < .001, η_*p*_^*2*^ = .25. Pairwise comparisons for each pair type demonstrated that identical pairs produced higher JOL ratings than similar pairs, *t*(25) = 3.90, *d* = 0.76, *p* = .004, but they did not significantly differ from related pairs, *t*(25) = 1.16*, d* = 0.23, *p* = 1.00. Importantly, identical pairs produced significantly higher JOLs than rotated pairs, *t*(25) = 4.05, *d* = 0.79, *p* = .003. Moreover, rotated pairs did not differ from related and similar pairs [*rotated* versus *similar: t*(25) = 0.80, *d* = 0.16, *p* = 1.00, *rotated* versus *related: t*(25) = 2.05, *d* = 0.40, *p* = .304]. Finally, related pairs produced higher JOLs than similar pairs, *t*(25) = 4.51, *d* = 0.88, *p* < .001.

#### Conditional Cued 4-AFC Recognition

Mean accuracy rates were submitted to a single-factor repeated measures ANOVA yielding a significant main effect, *F*(3,75) = 19.15, *MSE* = .015, *p* < .001, η_*p*_^2^ = .43. Pairwise comparisons showed that identical pairs produced lower accuracy rates than similar and related pairs [*identical* versus *similar: t*(25) = 4.71, *d* = 0.92, *p* < .001, *identical* versus *related: t(*25) = 4.19, *d* = 0.82, *p* = .002], but not different than rotated pairs, *t*(25) = 1.95, *d* = 0.38, *p* = .374. Rotated pairs produced lower accuracy than similar and related pairs [*rotated* versus *similar: t*(25) = 5.72, *d* = 1.12, *p* < .001, *rotated* versus *related: t*(25) = 6.17, *d* = 1.21, *p* < .001]. Finally, the accuracy rate of similar and related pairs did not differ, *t*(25) = 0.19, *d* = 0.04, *p* = 1.00.

#### Mediational Analysis

Experiment 3 used similar, rotated, identical, and related items, with “similar” items as the reference group. Except for the unrelated items replaced with rotated items, the analysis was identical with Experiment 2.

##### Identical Versus Similar Pairs

For identical pairs, the indirect effect through fluency was significant (β = 2.42, 95% CI [0.65, 4.20]), as was the direct effect (β = 9.73, 95% CI [5.91, 13.55]). The total effect was β = 12.14, 95% CI [8.92, 15.42], with 19.9% of the effect mediated by fluency (95% CI [0.05, 0.38]). The remaining 80.1% can be attributed to theory-based processes.

##### Rotated Versus Similar Pairs

For rotated items, only the indirect effect via fluency was significant (β = 2.18, 95% CI [0.60, 3.82]); the direct effect was nonsignificant (β = −0.34, 95% CI [−4.04, 3.30]). The direct and indirect effects showed opposite patterns, making total effect and the proportion mediated uninterpretable, thus were not reported.

##### Related Versus Similar Pairs

For related items, both indirect and direct pathways were significant. The indirect effect via fluency was β = 0.67 (95% CI [0.21, 1.29]), and the direct effect was β = 6.69 (95% CI [3.35, 10.00]). The total effect was β = 7.37 (95% CI [4.10, 10.82]), with 9% of the effect mediated by fluency (95% CI [0.02, 0.21]) and over 90% of the effect attributed to theory-based expectations. Thus, related pairs appear to engage both fluency and theory-based processes, but theory-based processes played the dominant role.

##### Identical Versus Rotated Pairs

The difference in the indirect effects between identical and rotated items was significant (β = 0.24, 95% CI [0.04, 0.60]), as was the difference in direct effects (β = 10.07, 95% CI [6.98, 13.35]). The total effect was β = 10.30, 95% CI [7.04, 13.38]. However, the difference in proportion mediated was not statistically reliable (β = 0.98, 95% CI [−2.10, 83.66], showing that fluency-based processes played a minimal role in boosting the JOLs for the identical pairs.

### Discussion

The results replicated Experiments 1 and 2, producing a metacognitive illusion for the identical pairs. Although identical pairs received the highest JOLs, they did not produce the highest memory performance. Moreover, even though rotated items showed the highest similarity to identical items in terms of both perceptual and conceptual resemblance, the hypothesized linear relationship between perceptual similarity and JOLs was not present in the current study, shown through mediational analyses. When similar pairs were compared to identical pairs, rotated pairs, and related pairs, the proportion mediated by RT, a measure indicative of fluency, either mediated a very low proportion of the relationship between item type and JOL, or it did not mediate the relationship significantly. For rotated items, which are visually degraded but still conceptually the same with identical items, JOLs appeared to be driven almost exclusively by theory-based processes, with little contribution from processing fluency. These results suggest a robust confidence boost for identical pairs that cannot be explained by reaction time through increased perceptual similarity.

## General Discussion

This study investigated whether the *identical effect*—higher judgments of learning (JOLs) but lower memory performance for identical pairs—extends to pictorial materials and explored its underlying mechanisms. Across three experiments, participants consistently gave higher JOLs to identical image pairs than to similar, related, or unrelated pairs. However, memory performance for identical pairs was either comparable to or lower than that for related and similar pairs. These results indicate that the metacognitive illusion observed with verbal stimuli also occurs with images, suggesting a parallel illusion in the visual domain.

The current study methodologically offers new insights into the underlying mechanisms of the identical effect. First, investigating the identical effect with pictorial stimuli allows for a more nuanced disentanglement of the contributions of conceptual and perceptual fluency in the identical effect. Initially, Castel et al. (2007) proposed that the higher JOLs for identical compared to related items could be attributed to processing fluency, driven by perceptual similarities between item pairs. However, their study did not directly assess the influence of perceptual similarities, potentially confounding them with conceptual similarities. As a follow-up, Mueller et al. (2016) aimed to isolate the impact of perceptual fluency by using alternating cases for identical words (e.g., dOG–DoG), thereby keeping conceptual aspects constant while reducing perceptual fluency. While this design may seem reasonable, participants might not attend to perceptual similarities (or differences) as much due to the automaticity of reading (Logan, 2006; MacLeod, 1991), potentially undermining the effectiveness of the alternating font manipulation. In the current study, participants encoded identical, similar, related, and unrelated image pairs providing us with a unique opportunity to investigate the role of perceptual similarities on JOLs without the interference of the reading process. When presented with two identical images of a dog, both conceptual and perceptual similarities (or sameness) are evident. Nevertheless, when two similar images of dogs are used, they remain conceptually identical but exhibit lower perceptual similarity than exact copies, allowing us to finesse the contribution of perceptual fluency in the identical effect. Thus, employing similar exemplar images as stimuli provided a natural gradient between identical and related items in terms of decreasing perceptual fluency while maintaining equivalent conceptual similarity, which is unique to pictorial materials. Finally, using related images (i.e., one cat and one dog), which display the lowest perceptual similarity compared to similar and identical items, enables us to observe the gradual changes in the perceptual characteristics of the pairs further. If the findings were a consequence of perceptual fluency and the natural degradation between similar versus related item pairs, the results should have shown gradually decreasing JOLs as the item pairs went from similar to related. Yet, the findings consistently revealed that this was not the case. Indeed, in Experiment 3, JOLs for related items were significantly higher than similar items. This finding clearly argues against the experience-based mechanisms of the identical effect. Moreover, the findings indicated that participants made faster judgments for identical compared to similar items. However, the extent to which this reaction time difference mediated the identical effect was minimal in Experiment 2. This finding challenges the notion of perceptual fluency as the primary driver of inflated JOLs for identical items, aligning with the conclusions drawn by Mueller et al. (2016) despite the differences across these two studies for fluency measures and methodological approaches.

Another distinctive aspect of the current study was the introduction of a rotation manipulation (Experiment 3). While previous studies have employed various manipulations, rotation offers a unique opportunity to further dissect the role of perceptual similarities in the identical effect. When an item is presented alongside its rotated counterpart, both conceptual and perceptual similarities to the target image remain intact, except for the orientation. Consequently, compared to similar and related images, the rotation condition should elicit faster reaction times and higher JOLs, indicating a contribution of perceptual fluency to the identical effect. However, the results of the current study suggest a different outcome. Despite being identified more rapidly than similar and related images, the rotated images actually yielded lower JOLs, challenging the notion that processing fluency singularly drives the identical effect. This finding was further supported by mediational analyses, which confirmed that processing fluency alone is not the determinant factor of the effect.

From a theoretical aspect, this study allows us to investigate the contribution of fluency versus beliefs to the identical effect more closely. As also explained before, the underlying mechanisms for making JOLs are typically hypothesized to fall into one of two categories: experience-based nonanalytic processes versus theory-based analytic processes. Both Experiments 2 and 3 showed repeatedly that the time taken to identify the type of relationship between the item pairs, which is typically associated with experience-based processes, is not linearly associated with the JOLs that participants provide. It instead varies with the degree of perceptual similarity. While processing fluency does have an effect, it is not consistently dominant. In cases where perceptual differences are subtle—such as between similar and identical pairs—participants still rely on theory-based reasoning, and the impact of fluency decreases. This subtle distinction is a key contribution of the present study, made possible by using visual rather than verbal materials.

Finally, the current study employs more appropriate statistical analyses to examine the role of processing fluency in the identical effect. Previous studies primarily relied on correlational analyses (e.g., Mueller et al., 2016), which, while informative, do not fully capture mediation effects. In contrast, this study uses mediation analyses with the Sobel test and bootstrapping methods, which provide greater statistical power to detect indirect effects when sample sizes and effect sizes are sufficient. These approaches allow for a more precise evaluation of the contribution of processing fluency by accounting for potential mediator variables (Hayes & Preacher, 2014). Clearly, these conclusions are drawn from intricate mediation analyses conducted with relatively small sample sizes, which raises the possibility that some observed effects may reflect Type I or Type II errors—even though we employed bias-corrected and accelerated (BCa) bootstrapping with 5,000 resamples to enhance robustness (MacKinnon et al., 1995). Further studies with larger samples are warranted to replicate these findings.

Another aspect of the current experiment concerns the accuracy rates of memory performance. Specifically, identical pairs exhibited similar recognition accuracy rates to related and similar pairs while demonstrating higher memory performance than unrelated pairs, thus confirming the presence of a metacognitive illusion. This finding contrasts with the typical pattern observed with verbal materials, where related images often yield higher memory performance than identical items. This usual pattern was only evident in Experiment 3 of our study and not in Experiments 1 and 2. Several factors may account for this discrepancy. First, the distinctiveness of pictorial stimuli may inherently lead to higher memory performance, potentially resulting in a ceiling effect. Despite increasing the number of item pairs from 40 to 80 trials in Experiment 2 and observing lower accuracy rates, the pattern persisted, refuting this possibility. Second, the composition of the encoding list may influence accuracy rates. Experiment 3 replaced unrelated items with rotated ones, which may lead to more source confusion for participants: When participants cannot recollect the target item, they may have difficulty assigning the responses to identical versus rotated targets, which produces a pattern that is more similar to those observed with verbal materials. Moreover, our encoding manipulation differed from previous studies by Castel et al. (2007) and Mueller et al. (2016). While they instructed participants to study items at their own pace passively, we required participants to assess the cue–target relationship between items, akin to the approach by Halamish and Undorf (2023). This active encoding process may have contributed to higher accuracy rates overall. Additionally, Halamish and Undorf (2023) found that making JOLs at encoding facilitated memory performance for identical and related pairs but not unrelated pairs, suggesting that the effort used in making these judgments may play a role.

A noteworthy observation in the current study concerns the reversal of the illusion of the competence effect. While Castel et al. (2007) demonstrated that participants overestimated their memory performance, the present results reveal the opposite pattern: Participants consistently underestimated their performance, leading to an *illusion of incompetence*. One explanation for this reversal could involve ceiling effects. When pictures are used, participants can encode items both visually and verbally, potentially inflating memory performance (Paivio, 1990). Another contributing factor might be the type of test employed. Unlike previous studies, which primarily used cued-recall tests suitable for verbal material, the current study used a cued 4-AFC recognition to accommodate the varied naming of pictorial material and to reduce ambiguity. Recognition tests may enhance memory sensitivity and produce higher accuracy. Interestingly, when the current JOLs are compared with those in Mueller et al., the absolute values across item types are similar. This suggests that participants may not fully account for differences in encoding format (visual vs. verbal) or test type (cued-recall vs. cued recognition) when making their JOLs, potentially contributing to the observed reversal.

In summary, this study extends the findings of the identical effect from verbal to pictorial materials and challenges the notion that perceptual similarities between pairs are the sole driving factor for boosted JOLs for the identical pairs. Instead, various belief-based processes or conceptual relatedness between item pairs may underlie this relationship. Even though processing fluency plays a role in the process, a priori beliefs about the effects of the manipulation may be the dominant factor in making JOLs. Further investigations are warranted to explore these issues in greater depth.

## Footnotes

^1^
Recognition accuracy analyses were conducted on the data set after excluding trials that were incorrectly identified with respect to pair type during the encoding phase of both Experiments 2 and 3. The same data were also excluded for the mediational analyses.

## Notes

**Publication Ethics:** Informed consent was obtained from all participants included in the study. All the procedures for the studies reported in this paper were approved by the Bilkent University Ethics Committee (Approval #2015_03_18_05).

**Open Science:** **Open Code:** The coding scheme for Experiments 1–3 are available online at the following link: https://osf.io/mxgsd/ (Filiz & Besken, 2025).

**Open Data:** The data for Experiments 1–3 are available online at the following link: https://osf.io/mxgsd/ (Filiz & Besken, 2025).

**Open Materials:** Only instructions for the experiments are available online as materials (https://osf.io/mxgsd/), because some of the materials used are adapted from Kovalenko et al. (2012) and are copyright protected.

**Preregistration:** The experiments were not pre-registered.
